# Identification of Causal Genes of COVID-19 Using the SMR Method

**DOI:** 10.3389/fgene.2021.690349

**Published:** 2021-07-05

**Authors:** Yan Zong, Xiaofei Li

**Affiliations:** Department of Infectious Diseases, Yiwu Central Hospital, Jinhua, China

**Keywords:** SMR, COVID-19, eQTL, GWAS, UQCRH, PPA2, OGT, PANO1

## Abstract

Since the first report of COVID-19 in December 2019, more than 100 million people have been infected with SARS-CoV-2. Despite ongoing research, there is still limited knowledge about the genetic causes of COVID-19. To resolve this problem, we applied the SMR method to analyze the genes involved in COVID-19 pathogenesis by the integration of multiple omics data. Here, we assessed the SNPs associated with COVID-19 risk from the GWAS data of Spanish and Italian patients and lung eQTL data from the GTEx project. Then, GWAS and eQTL data were integrated by summary-data-based (SMR) methods using SNPs as instrumental variables (IVs). As a result, six protein-coding and five non-protein-coding genes regulated by nine SNPs were identified as significant risk factors for COVID-19. Functional analysis of these genes showed that UQCRH participates in cardiac muscle contraction, PPA2 is closely related to sudden cardiac failure (SCD), and OGT, as the interacting gene partner of PANO1, is associated with neurological disease. Observational studies show that myocardial damage, SCD, and neurological disease often occur in COVID-19 patients. Thus, our findings provide a potential molecular mechanism for understanding the complications of COVID-19.

## Introduction

In December 2019, SARS-CoV-2 was first reported to lead to the respiratory disease coronavirus disease 2019 (COVID-19) (Zhou et al., 2020). Subsequently, COVID-19 quickly spread to all parts of the world and became a worldwide public health event. As of February 17, 2021, more than 100 million people had been infected, and more than 2.4 million people had died of COVID-19. At present, a total of seven types of coronaviruses that can infect humans have been discovered, including SARS-CoV, SARS-CoV-2, and MERS-CoV, which have high case fatality rates (CFRs) (Gussow et al., 2020). The other four coronaviruses, HCoV-HKU1, HCoV-NL63, HCoV-OC43, and HCoV-229E, only cause mild symptoms in humans. Although the CFR of SARS-CoV-2 is relatively lower than those of SARS-CoV and MERS-CoV, it is still highly infectious.

Exploring the origin of the virus would help to increase the understanding of SARS-CoV-2 (Cui et al., 2019; Narang et al., 2019; Benetti et al., 2020; Meng et al., 2020; Wan et al., 2020; Qi et al., 2021). Previous studies have shown that bats are the natural host of the evolved coronavirus (Jiang et al., 2020; Kwon et al., 2020). Based on the alignment of the reference genome sequence, a phylogenetic tree was constructed, indicating that the genes are very similar between SARS-CoV-2 and members of the bat Sarbecovirus subgenus Betacoronavirus (Wu et al., 2020). According to sequence mapping, the whole-genome sequence of SARS-CoV-2 has the highest similarity with SARS-CoV BatCoV RaTG13, reaching over 96%. The similarity between SARS-CoV-2 and the coronaviruses SARS-CoV and MERS-CoV is only 79 and 50%, respectively (Andersen et al., 2020). Although the specific route of transmission of SARS-CoV-2 from its natural host to humans is not yet clear, researchers have discovered that the key functional sites of the SARS-CoV-2 spike protein are almost the same as those of the virus isolated from pangolins. Therefore, a pangolin coronavirus may have provided part of the spike gene for SARS-CoV-2.

Computational methods with omics data have shown strong power in identifying disease-related genes (Zhao T. et al., 2020c). SARS-CoV-2 is a single-stranded RNA virus (Chen et al., 2020). The reference genome shows that it has 29,903 nucleic acid base pairs, including seven conserved unstructured protein domains and four structural protein domains, including the spike protein. The sequence of SARS-CoV-2 has mutated since its discovery. As early as March 2020, researchers analyzed 160 early virus strains and found three important single-point mutations, T8782C, C28144T, and G26144T (Forster et al., 2020). Among them, the mutations T8782C and C28144T are used to distinguish between type A and B viruses, and the mutation G26144T represents a newer virus type (type C). In May 2020, Cheng et al. analyzed more than 1,800 strains of viruses in the Americas, Europe, and Asia and verified that type B viruses are more infectious and have almost replaced the type A viruses in current circulation (Cheng et al., 2021). Through comparison with the reference genome, it was found that each virus mutated at approximately 1.75 sites per month. The overall mutations were silent mutations and would not cause major functional changes in the virus. Cheng et al. proposed to analyze the mutation rules of the virus over time and identified seven dominant mutations. According to functional bioinformatics analysis, these mutations likely caused the virus to decrease in toxicity and increase in infectivity. In addition, mutation clustering information (Zhu X. et al., 2019; Qi et al., 2020; Zhao T. et al., 2020b; Zhao X. et al., 2020; Zou et al., 2020; Zhao et al., 2021) indicated that the virus circulating in the Americas is more similar to RaTG13.

By searching for the origin of SARS-CoV-2, researchers also found that SARS-CoV-2 and SARS-CoV use the same receptor, angiotensin-converting enzyme II (ACE2). SARS-CoV-2 invades human cells by binding to ACE2. By interacting with proteins in human cells, SARS-CoV-2 affects human health. In August 2020, researchers found hundreds of interactions between human proteins and SARS-CoV-2 proteins. Although many proteins that interact with SARS-CoV-2 have been discovered so far, the genes associated with SARS-CoV-2 pathogenesis are still unknown. Since genomics data of many COVID-19 patients have been reported, there is an opportunity to find potential risk genes through analysis and integration of omics data of susceptible populations (Cheng, 2019; Cheng et al., 2019; Li F. et al., 2020; Wang et al., 2020).

SMR is a method used to determine the causal association between genetically determined traits and diseases, and eQTLs are genetic variations related to the expression of traits. Since eQTL data are tissue specific, it is possible to correlate the eQTL data of disease-related tissues with disease GWAS data and use the SMR method to find causal genes for diseases. Therefore, in our study, we used lung eQTL data of GTEx and GWAS data of COVID-19 patients to identify the genes related to COVID-19 pathogenesis using the SMR method.

## Materials and Methods

### Risk SNPs for Severe COVID-19

**FIGURE 1:**
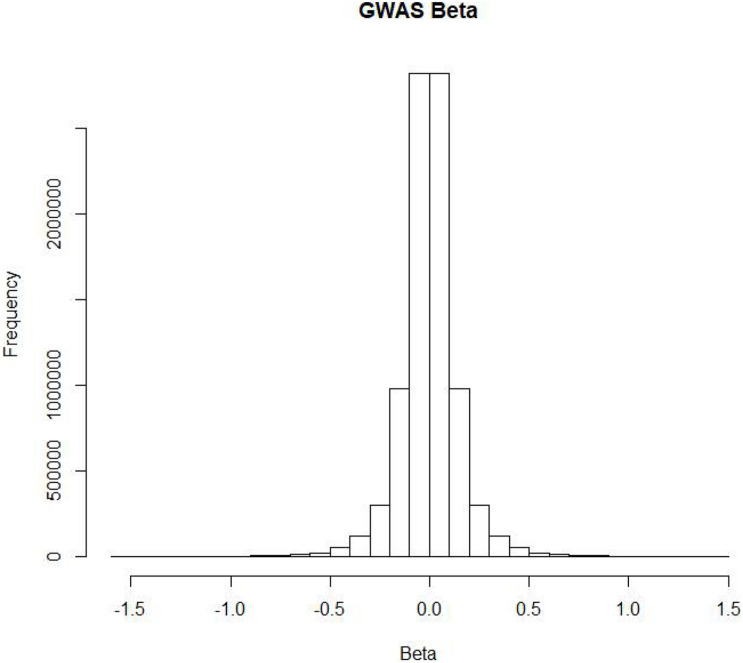
The distribution of GWAS beta in severe COVID-19 patients.

According to current knowledge, some people are more susceptible than others to COVID-19. To assess the impact of SNPs on the risk of COVID-19, researchers conducted a genome-wide association study (GWAS) on two groups of European patients in seven hospitals in Italy and Spain and performed a meta-analysis of the results of the two groups (Ellinghaus et al., 2020). First, quality control was conducted on 1980 severe COVID-19 patients, and 1,610 patients remained after removing population outliers. Then, the researchers performed a GWAS on 835 Italian patients and 1,255 control group members (Sun et al., 2019), as well as 775 Spanish patients and 950 control group members. As a result, they obtained severe COVID-19 risk data for 8,582,968 SNPs. Finally, the two sets of experimental results were meta-analyzed to obtain the final risk SNPs. Figure 1 shows the distribution of risk betas of SNPs in COVID-19 patients. Most SNPs had no impact on the risk of COVID-19. In addition, researchers found that COVID-19 high-risk SNPs clustered on 3p21.31.

### Lung eQTL

**FIGURE 2:**
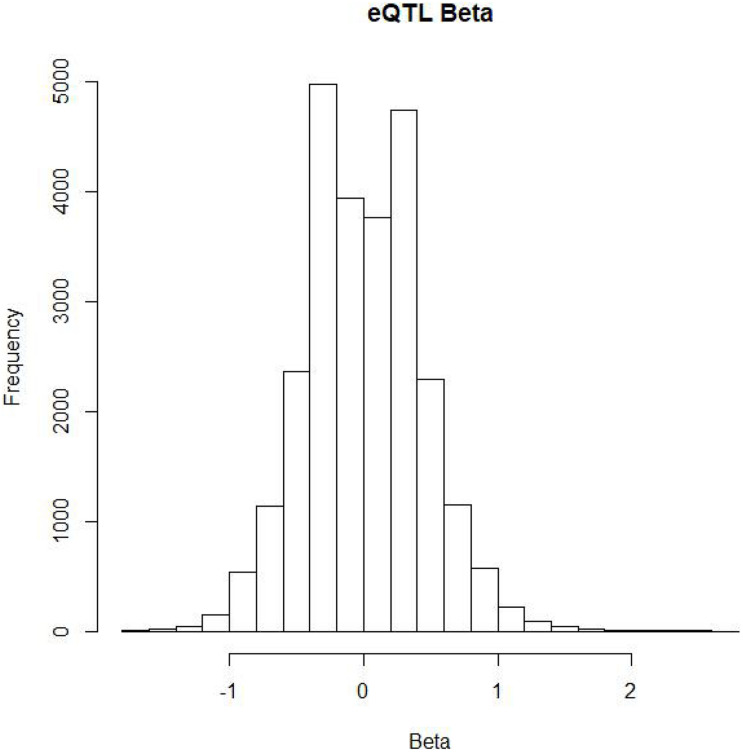
The distribution of eQTL beta on lung SNPs.

To date, many GWAS have been performed. These studies have identified thousands of disease-related risk SNPs. Since more than 90% of SNP sites exist outside of protein-coding genes, it is difficult to understand the mechanisms by which these SNPs affect diseases. To this end, researchers investigate expressed quantitative trait loci (eQTLs) to reveal the genes regulated by SNPs in the blood, lung and other tissues (Zhao T. et al., 2020a). Therefore, the NIH launched the gene type-tissue expression (GTEx) project, with the goal of establishing the relationships between SNPs and gene expression in different tissues. Currently, the project has accepted more than 900 post-mortem donors. Sequencing of different tissues in the donors has identified a large number of SNP-regulated genes. Summarized GTEx data could be obtained from the project’s website. Figure 2 shows the distribution of beta values of lung SNPs on gene expression.

### Identification of Potential Causal Genes of COVID-19 Based on the SMR Method

In the domain of biomedicine, many causal disease associations have been discovered through observational research, such as the association between smoking and lung cancer (Ghosh and Yan, 2020; Li J. et al., 2020; Yuan et al., 2020). However, the associations between phenotypes and diseases found in these observational studies cannot reflect causality. However, because observational studies are usually disturbed by external factors and often face practical problems related to long time frames and high costs, there are large errors in the analysis of pathogenic factors of diseases. Mendelian randomization follows the Mendelian inheritance law of allele separation and free recombination of nonalleles and makes causal inferences based on genetic variation, which does not change with environment or age, so this method is widely used in causal inference of pathogenic factors. Sometimes, due to the influence of confounding factors, the correlation found is not accurate. This greatly limits the development of the field. To solve this problem, the statistician Katan (1986) introduced the concept of Mendelian randomization (MR) in 1986 to study whether low serum cholesterol levels can increase the risk of cancer. MR uses genotype as an instrumental variable (IV) and applies the two-stage least squares method to infer the pathogenicity of diseases. With the gradual deepening of GWAS research, this method has been widely applied using SNPs as IVs. This method assumes that Z is an instrumental variable (SNP), X represents exposure factors or gene expression levels, and Y represents the disease. According to the two-stage least squares method, the effect of X on Y is evaluated as follows.

(1) $$B\,e\,t\,a_{X\,Y}=B\,e\,t\,a_{Z\,X}/B\,e\,t\,a_{Z\,Y}$$

Here, *Beta*_*ZX*_ represents the least-squares estimate of *Z* on *X*, and *Beta*_*ZY*_ is the least-squares estimate of *Z* on *Y*. Then, we estimate the significance of *X* on *Y* as *T*_*MR*_.

(2) $$T_{M\,R}={\left(B\,e\,t\,a_{X\,Y}\right)}^{2}/v\,a\,r\,\left(B\,e\,t\,a_{X\,Y}\right)$$

Theoretically, GWAS and eQTL need to target the same sample, but GWAS and eQTL are performed independently, so we use the two-sample MR method, and *T*_*SMR*_ is obtained as the final evaluation value (Zhao S. et al., 2019; Zhao T. et al., 2019):

(3) TS⁢M⁢R=ZZ⁢Y2⁣*⁢ZZ⁢X2/(ZZ⁢Y2⁢ZZ⁢X2)

where Zzy represents the Z statistics from the GWAS, and Zzx represents the z statistics from the eQTL study. Since the true values of *Beta*_*ZX*_ and *Beta*_*ZY*_ cannot be obtained, we can use estimations to replace them. The *T*_*SMR*_ yields an approximate Chi-square test statistic.

**FIGURE 3:**
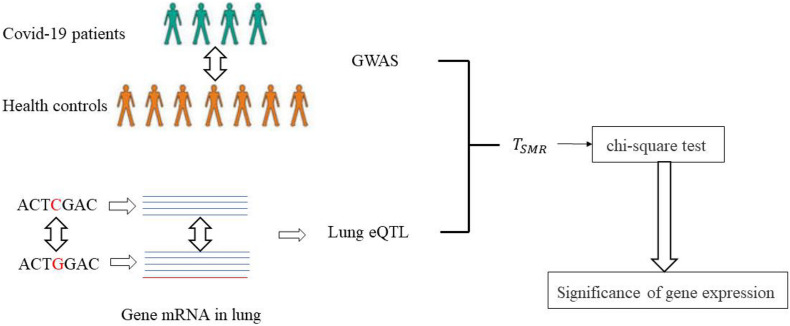
The workflow of identifying causal genes of COVID-19 using the SMR method.

Here, we used the SMR method to evaluate the mechanism by which SNPs affect COVID-19 and identify potential pathogenic genes in the lungs. The specific process is shown in Figure 3. The GWAS data of COVID-19 patients and the lung eQTL data were obtained from a public dataset. *T*_*SMR*_ was calculated based on the two-sample MR method, which was then further evaluated using the chi-square test to identify significant SNPs and genes.

## Results

### Causal SNPs and Genes of COVID-19

**FIGURE 4:**
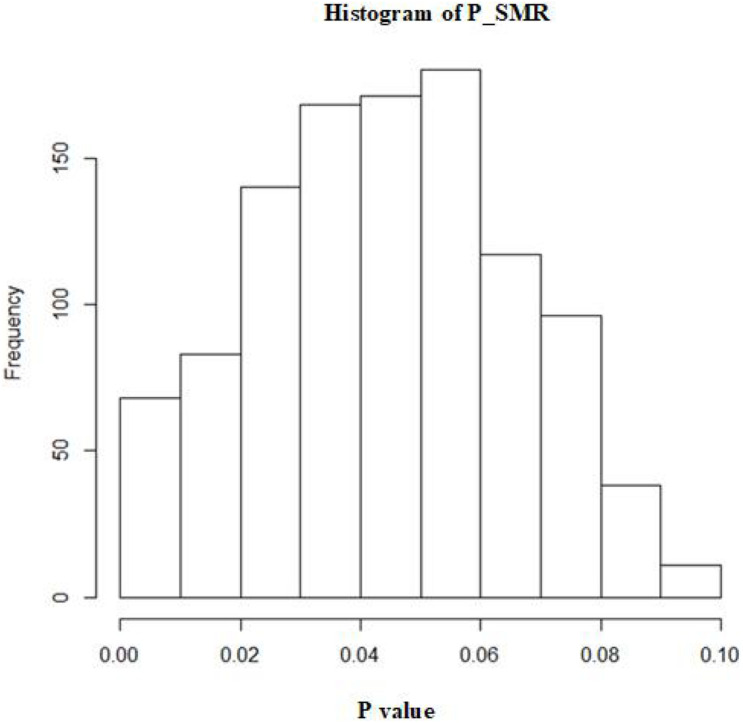
The distribution of SMR *p*-values of SNPs.

**FIGURE 5:**
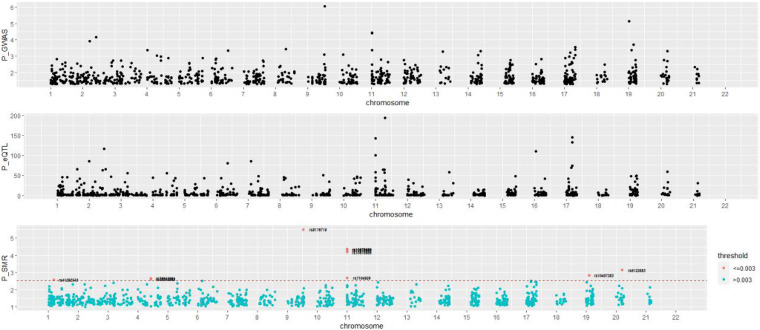
The experimental results based on SMR.

A total of 1,072 SNPs appeared in both GWAS and eQTL data. After application of the SMR method, 1,072 SNPs were determined to regulate the P value distribution of COVID-19 through genes (Figure 4). The P values of SMR for most SNPs were concentrated in the range of 0.02–0.08. Here, the threshold was set as 0.003, and then 11 genes (UQCRH, PPA2, PAPSS1, ABO, AP006621.5, CMB9-55F22.1, AP006621.6, PANO1, CTD-2027I19.2, LINC01273, and ARSA) regulated by nine SNPs (rs41292543, rs35258888, rs70947091, rs8176719, rs10678686, rs10678686, rs10678686, rs7104929, rs10407383, rs6122883, and rs6151429) with P values lower than the threshold were considered to increase the risk of COVID-19 (Table 1). Figure 5 shows the GWAS P value, eQTL P value, and SMR *P*-value of all SNPs.

**TABLE 1:** Nine Causal SNPs and eleven pathogenic genes of COVID-19.

SNP	P_SMR	Gene	chr	pos	P_GWAS	P_eQTL
rs41292543	0.002574	UQCRH	1	46309111	0.001553	2.65*E*−16
rs35258888	0.002419	PPA2	4	105355205	0.001035	2.61*E*−10
rs70947091	0.002154	PAPSS1	4	107694523	0.001899	2.28*E*−56
rs8176719	3.07E-06	ABO	9	133257521	8.76*E*−07	1.27*E*−34
rs10678686	4.68E-05	AP006621.5	11	780321	3.96*E*−05	1.28*E*−100
rs10678686	4.34E-05	CMB9-55F22.1	11	780321	3.96*E*−05	9.62*E*−143
rs10678686	6.40E-05	AP006621.6	11	780321	3.96*E*−05	1.12*E*−44
rs7104929	0.001973	PANO1	11	784340	3.73*E*−05	0.0111629
rs10407383	0.001423	CTD-2027I19.2	19	24134099	0.000412	4.61*E*−09
rs6122883	0.000702	LINC01273	20	50172836	0.000494	3.43*E*−34
rs6151429	0.000645	ARSA	22	50625049	0.000522	4.70*E*−50

### Functional Analysis of Causal Genes

Among the identified pathogenic genes, there were a total of six protein-coding genes (UQCRH, PPA2, PAPSS1, ABO, PANO1, and ARSA) and five noncoding genes. We then performed functional analysis of these six protein-coding genes to identify their functions, related diseases and pathways.

UQCRH participates in cardiac muscle contraction. A large number of studies have found that approximately 8–12% of COVID-19 patients have myocardial damage (Lippi et al., 2020). Although heart problems are usually not the most prominent or deadly feature of COVID-19, they are common and are severe enough that most people admitted to the hospital for COVID-19 are now being screened for myocardial damage. There are many potential causes of COVID-19-related myocardial damage, but it is often difficult to determine the specific cause in a specific individual. The UQCRH gene found here may be a potential cause. In addition, according to Gene Ontology annotation, UQCRH has ubiquinol-cytochrome-c reductase activity.

PPA2 is closely related to sudden cardiac failure (SCD). At present, there have been reports of COVID-19 patients who have died of SCD. In July 2020, Samira et al. diagnosed three patients with COVID-19 according to the reverse transcriptase-polymerase chain reaction of nasopharyngeal swabs and radiological examinations (Shirazi et al., 2021) who eventually died of SCD. Thus, the authors recommended that it is necessary to monitor the heart conditions of COVID-19 patients. Although there is no direct causal link between SCD and COVID-19, analysis of current data shows that there is a reasonable link between them. According to the latest studies, the incidence of SCD in the community and hospital environment has increased since the outbreak of COVID-19 (Yadav et al., 2020). Based on our findings, PPA2 can increase the risk of COVID-19, so PPA2 may be a potential factor that results in SCD in COVID-19 patients.

### Interaction Between the Causal Gene PANO1 and OGT

We searched for the interactions between causal genes of COVID-19 and other genes. As a result, we found that OGT can interact with PANO1. Then, we further investigated the function of OGT to explore the potential mechanism of PANO1 in the risk of COVID-19. At the start of the COVID-19 epidemic, some patients experienced neurological symptoms, such as feeling confused, being unable to discern direction, and feeling restless (Marshall, 2020). A total of 0.2% of the patients of two other SARS-CoV-2-related coronaviruses, SARS-CoV and MERS-CoV, have neurological disease. Given the number of COVID-19 patients, hundreds of thousands of patients may have neurological complications. As genes related to neurological disease and risk genes for COVID-19, OGT, and PANO1 must be considered further.

## Conclusion

In this article, we used the SMR method to analyze the genes involved in COVID-19 pathogenesis. Here, the risk SNPs for COVID-19 were derived from the GWAS data of Spanish and Italian patients. Lung eQTL data were acquired from the GTEx project. In the postgenomic era, MR and SMR methods have been widely used (Liu et al., 2018). Currently, a large number of pathogenic phenotypes and genes have been identified based on these methods. Through SMR, this article discovered six protein-coding genes and five noncoding genes that can increase the risk of COVID-19. Finally, nine SNPs that met the threshold conditions were identified, and the SMR method was used to determine that these SNPs regulated 11 disease-causing genes that could increase the risk of COVID-19. Then, disease pathway enrichment analysis was performed on these genes.

Through functional analysis, we found that UQCRH participates in cardiac muscle contraction, PPA2 is closely related to SCD, and myocardial damage and SCD occurred in patients with COVID-19. Therefore, our findings provide a potential molecular mechanism for these processes. Further analysis revealed an interaction between OGT and PANO1. OGT is associated with neurological disease. This may explain the neurological complications in COVID-19 patients.

## Data Availability Statement

The original contributions presented in the study are included in the article/supplementary material, further inquiries can be directed to the corresponding author/s.

## Ethics Statement

Ethical review and approval was not required for the study on human participants in accordance with the local legislation and institutional requirements. Written informed consent for participation was not required for this study in accordance with the national legislation and the institutional requirements.

## Author Contributions

YZ and XL wrote the manuscript and did the experiments. XL provided ideas of this work. YZ analyzed the data. Both authors approved the submitted version.

## Conflict of Interest

The authors declare that the research was conducted in the absence of any commercial or financial relationships that could be construed as a potential conflict of interest.
